# Challenges in the Clinical Diagnosis of Lithium Toxicity: A Case Report

**DOI:** 10.7759/cureus.47503

**Published:** 2023-10-23

**Authors:** Ahmad H Almadani, Fay H AlBuqami, Mohammed A Aljaffer

**Affiliations:** 1 Department of Psychiatry, College of Medicine, King Saud University, Riyadh, SAU; 2 College of Medicine, King Saud University, Riyadh, SAU

**Keywords:** saudi arabia, bipolar disorder, hyperammonemic encephalopathy, neuroleptic malignant syndrome, serotonin syndrome, syndrome of irreversible lithium-effectuated neurotoxicity, lithium toxicity

## Abstract

Lithium, a medication commonly used to treat bipolar disorders, has a narrow therapeutic index, putting patients at risk of lithium toxicity. Such toxicity could entail neurological-related complications and could be precipitated by several factors. In this paper, the authors discuss a case of a middle-aged woman taking lithium for bipolar disorder who presented to the emergency department with altered mental status, tremors, generalized weakness, and dysarthria. Multiple differential diagnoses were considered during her hospitalization, which included an admission to the intensive care unit. This case highlights the variability of lithium toxicity presentations and its management challenges. Further research is needed to understand such manifestations, potential precipitating factors, differential diagnoses, and effective detection and management.

## Introduction

Lithium is one of the commonly used mood stabilizers for bipolar disorder (BD) [1]. It was discovered in the nineteenth century, and it was introduced into therapeutic clinical practice in 1949 [2]. It is recommended as a first-line maintenance therapy for BD by various international medical guidelines such as the Canadian Network for Mood and Anxiety Treatments guidelines [3]. It has also been shown to prevent or ameliorate manic and depressive episodes of BD and reduce suicide risk [4,5]. The effective therapeutic ranges of lithium for BD differ based on several factors: indication for use (acute treatment vs. maintenance), the guidelines followed, and the patient’s age, among other factors [6].

Psychiatrists are cautious when using lithium, however, due to its narrow therapeutic index, which gives it the potential to result in toxicity [7]. Lithium toxicity may be the result of accidental or intentional overdose or intoxication for several reasons, including reduced renal clearance [7]. Lithium neurotoxicity is a spectrum of signs and symptoms that include confusion, mental slowing, dysarthria, mood changes, memory and executive dysfunction, seizures, and stupor [8-10].

In this case report, we describe the case of a patient who presented with a decreased level of consciousness (LOC) in the context of acute lithium toxicity.

## Case presentation

A woman in her sixties presented to the emergency department with progressively decreasing LOC for six weeks, along with dysarthria, intermittent tremors, generalized weakness, decreased oral intake, and two episodes of vomiting in the four days prior to her presentation. Her family reported noticing her exhibiting confusion, speech difficulties, decreasing LOC, tremors, rigidity, and uncontrollable voiding. Her medical history included history of dihydrolipoamide dehydrogenase deficiency, non-alcoholic fatty liver disease, and cholelithiasis.

The patient has had BD (type I) for 20 years. Seven weeks before her emergency room (ER) presentation, she was started on lithium carbonate (sustained-release preparation) 300 mg orally twice daily (BID), noting that before her ER presentation, the patient was following up with a psychiatrist outside the facility where she presented. Four weeks after the lithium initiation, the lithium level was drawn in the context of the patient’s partial response. The lithium level at that point was 0.52 mEq/L. After that, the lithium dosage was increased to 600 mg BID by her primary psychiatrist and then decreased again four days before her ER presentation to 900 mg, as the patient developed imbalance, loss of attention, and decreased oral intake. However, one day before her ER presentation, the patient’s family reduced the lithium dose to 300 mg, which, according to the documentation, was the last dose the patient took before her ER arrival. The patient was also on clozapine 400 mg once daily (OD), quetiapine 800 mg OD, and aripiprazole 10 mg OD. Following the lithium dosage increase (i.e., from 300 mg BID to 600 mg BID), the dosages of all these medications were reduced, with the intention of eventually replacing all of them with lithium. Specifically, clozapine was reduced to 200 mg OD, quetiapine to 600 mg OD, and aripiprazole to 5 mg OD. Prior to her ER presentation, there was no history of lithium toxicity or substance abuse.

**Figure 1:**
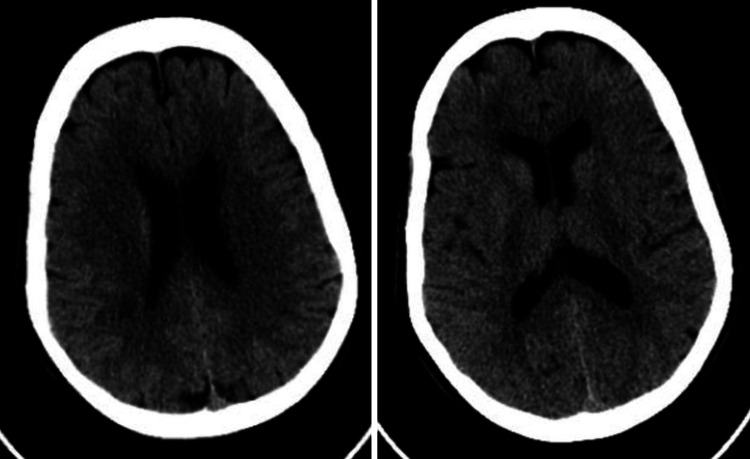
Unenhanced CT brain showing few focal hypodensities involving the right frontal and right parietal lobes

The physical examination in the ER revealed that the patient was drowsy with hypoactive reflexes. Her vital signs were as follows: respiratory rate of 18 breaths per minute, blood pressure 124/65 mmHg, oxygen saturation (SpO2) of 96%, and temperature of 36.8 °C. The neurological examination revealed slurred speech and hypoactive reflexes. The physical examination found no other systemic abnormalities. She had a Glasgow Coma Scale (GCS) score of 12/15 (eye-opening response (E) = 3; verbal response (V) = 4; motor response (M) = 5). The Initial work-up showed that the lithium level was high. Table 1 shows the laboratory test results. A plain cranial CT scan was performed, but the results were found to be unremarkable (Figure 1).

**Table 1:** Laboratory test results in the ER

Laboratory Test	Result	Normal Range
White Blood Cells	10.5 x10^9/L	4.0-11.0 x10^9/L
Red Blood Cells	3.9 x10^12/L	4.2-5.5 x10^12/L
Hemoglobin	127 gm/L	120.0-160.0 gm/L
Hematocrit	35.5%	37-47%
Mean Corpuscular Volume	90.1 fL	80.0-94.0 fL
Mean Corpuscular Hemoglobin	32.5 pg	27.0-32.0 pg
Mean Corpuscular Hemoglobin Concentration	361.0 gm/L	320.0-360.0 gm/L
Red Cell Distribution Width	13.3%	11.5-14.5%
Platelets	152.0x10^9/L	140.0-450.0 x10^9/L
Mean Platelet Volume	8.7 fL	7.2-11.1 fL
pH	7.39	7.34–7.45
Partial Pressure of Carbon Dioxide (PaO_2_)	38 mmHg	41–54 mmHg
Partial Pressure of Bicarbonate (HCO_3_)	23 mmHg	20–24 mmHg
QTC Interval	430 ms	360–460 ms
Creatinine Level	125 μmol/L	45–84 μmol/L
Blood Urea Nitrogen	10 mmol/L	2.76–8.07 mmol/L
Total Bilirubin	12.1 µmol/L	3.0-17 µmol/L
Lithium Level	3.20 mEq/L	Varies depending on multiple factors, such as the patient’s age and the stage of the illness.

**Figure 2:**
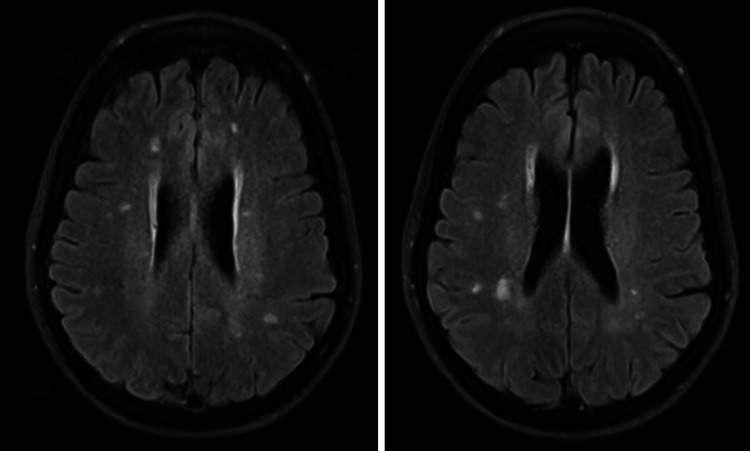
MRI Brain (T2/FLAIR) showing multiple scattered foci of T2 hyperintensity in the frontoparietal white matter and left temporal lobe, nonspecific in appearance and distribution FLAIR: fluid-attenuated inversion recovery

The patient was then admitted to the general internal medicine ward. The psychiatric consultation liaison team was involved early on, and the team recommended that all the psychotropic medications be held, as the initial impression was acute lithium toxicity. In the medical ward, the patient was initially managed with normal saline at 150 mL/hr (sodium chloride 0.9%). Her renal panel indicated acute renal injury. The electrocardiogram was normal, with no ischemic or arrhythmic changes and a normal QTC interval. The nephrology team was consulted for dialysis assessment, which proved unnecessary due to lithium levels decreasing from 3.2 mEq/L to 2.16 mEq/L within 24 hours. Despite the decrease in lithium serum levels, the patient’s LOC showed no improvement, nor did her GCS score. Magnetic resonance imaging (MRI) was performed to rule out structural causes, and it showed nonspecific white matter changes (Figure 2).

On the third day following her admission, the physical examination revealed cogwheel rigidity in the upper limbs and neck, brisk reflexes, and sustained clonus all over, with resting tremors mainly in the right upper arm and lower limbs. The GCS score was 8/15 (E2, V2, M4). The lithium level reached 0.58 mEq/L. The repeated lab results were aligned with normal hyperchloremic anion gap metabolic acidosis. Neuroleptic malignant syndrome (NMS) and serotonin syndrome (SS) were considered differential diagnoses. The electroencephalogram (EEG) showed a generalized slowing of the background activity, with occasional spike waves in the frontal region, possibly indicative of nonconvulsive status epilepticus (NCSE).

The patient was then shifted to the intensive care unit (ICU) for continuous electroencephalographic monitoring (cEEG) due to her NCSE and for electrolyte monitoring and correction. She was switched to intravenous (IV) sodium bicarbonate (NaHCO_3_) along with sodium phosphate. To manage the ongoing seizure, she was given 1 mg of lorazepam intravenously, valproic acid 500 mg IV, and 100 mg of topiramate BID until she improved and her GCS score became 10/15. However, her condition was complicated by bacteremia; a blood culture was taken and found to contain positive cocci in clusters. Hence, tazobactam IV was given to manage the bacteremia. She was also kept on enoxaparin 40 mg as prophylaxis for deep venous thrombosis, and EEG monitoring was used to detect jerky movements.

On the tenth day following admission, while the patient was in the ICU, further laboratory tests were ordered (Table 2). Abdominal ultrasound was completed twice and was unremarkable. Clinically, the patient still had rigidity and was not moving her limbs (showing mild flex only), with no jerky movements apparent. She had increased coughing and secretions and was placed on nasal cannula 2 L due to desatting (Oxygen saturation (SpO_2_): 89-93%; normal range: 95-100%).

**Table 2:** Laboratory test results on the tenth day of admission

Laboratory Test	Result	Normal Range
Creatine Kinase	38 units/L	26–192 units/L
Aspartate Transaminase	27.80 unit/L	0–32 unit/L
Alanine Transaminase	66.2 units/L	0–33 units/L
Alkaline Phosphatase	368 units/L	35–104 units/L
Ammonia	90.8 µmol/L	11–51 µmol/L

**Figure 3:**
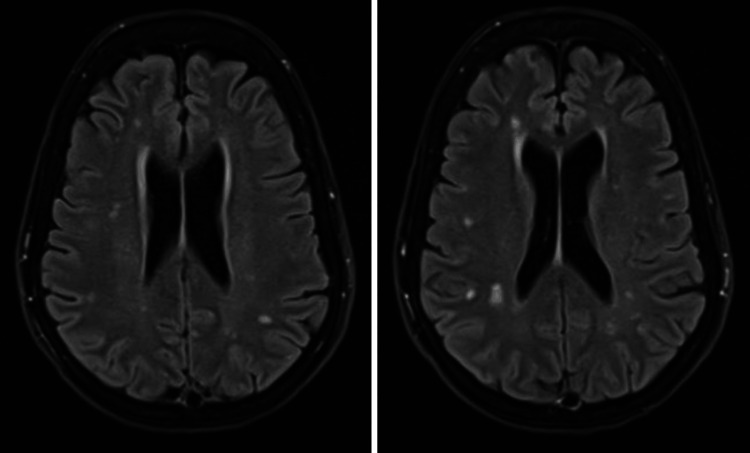
MRI brain (T2/FLAIR) showing unchanged multiple scattered foci of T2 hyperintensity in the frontoparietal white matter and left temporal lobe, nonspecific in appearance and distribution FLAIR: fluid-attenuated inversion recovery

The patient’s LOC showed no improvement, and this finding was thought to be likely multifactorial (secondary to the lithium toxicity, post-ictal state, anti-seizure medication (ASM), or hyperammonemia (HAE)). Table 3 shows the electrolyte profile of the patient. The patient had a disturbed electrolyte profile of hypokalemia, hypomagnesemia, and hypophosphatemia, which was managed with replacement therapy. MRI was repeated and showed unchanged non-specific white matter changes (Figure 3). During her ICU stay, the patient was found to be coronavirus disease 2019 (COVID-19)-positive. The EEG was repeated and revealed mostly triphasic waves mixed with delta and theta waves slowing, suggestive of metabolic encephalopathy with no clear electrographic seizures, and was deemed by the medical team to be less likely to be related to lithium toxicity or HAE. The ammonia level was tested again and returned high (119 µmol/L; normal range: 11-51). Lactulose 20 mL orally was started as management initially, followed by levocarnitine 250 mg started orally through nasogastric tube feeding, then 250 mg BID, and then 1 g. The patient was shifted from the ICU to the medical ward on day 12 after admission, amounting to 10 days of ICU stay.

**Table 3:** The electrolyte profile

Laboratory Test	Result	Normal Range
Sodium	149.38 mmol/L	136-145 mmol/L
Potassium	3.33 mmol/L	3.5-5.1 mmol/L
Chloride	113.26 mmol/L	98.0-107 mmol/L
Phosphorus	0.46 mmol/L	0.81-1.45 mmol/L

By day 17 after admission, as ammonia levels improved, the patient’s LOC began to improve, along with dysarthria and tremor. On day 24, the ammonia level returned to normal at 50.2 µmol/L (normal range: 11-51), the patient’s GCS reached 15/15 (M6, V5, E4), and the patient improved completely in terms of her LOC.

The patient was discharged from the hospital with the following medications: quetiapine 200 mg OD, atorvastatin 10 mg OD, esomeprazole 40 mg OD, l-carnitine 100 mg OD, and lactulose 15 mg OD. The discharge management plan also included physiotherapy (as she had weakness due to her ICU stay and immobilization) and a follow-up appointment within a week with the facility’s neuropsychiatric clinic to decide whether to resume her previous psychotropic medication regimen. Subsequently, the patient was seen in the neuropsychiatric clinic. Her condition was stable, with no remnant symptoms of the initial presentation. Moreover, the patient returned to her usual baseline of function, as reported by the family and found in the mental status examination. The patient also denied any manic or hypomanic symptoms, and the family reported no safety issues. The patient’s family, however, reported delusional ideas regarding the housemaid. Thus, the regular dose of quetiapine was increased from 200 mg to 300 mg, and the patient was prescribed an additional 100 mg as needed.

## Discussion

In the presented case, the initial diagnosis of lithium toxicity was established based on the patient’s clinical history and lithium serum levels (3.2 mEq/L). The patient’s management initially focused on fluid replacement and resolving the concurrent acute kidney injury. There was no need for hemodialysis, as deemed by the nephrology team. Lithium levels were normalizing at sufficient levels, from 3.2 mEq/L to 2.16 mEq/L within 24 hours. Of note, extracorporeal lithium removal is recommended if kidney function is impaired and the lithium serum level is more than 4 mEq/L or one of the following is present: decreased LOC, seizure, or life-threatening dysrhythmias, irrespective of lithium levels [11]. It is also suggested if lithium is more than 5.0 mEq/L, significant confusion is present, or the time expected to reduce lithium levels to less than 1.0 mEq/L is more than 36 hours [11].

Several differential diagnoses for the patient’s presentation were considered. These differentials included SILENT, NMS, SS, SARS-CoV-2, NCSE, and metabolic encephalopathy. In 1987, Adityanjee et al. suggested the acronym “SILENT” to describe persistent neurotoxic sequelae of lithium toxicity [12]. SILENT is a clinical diagnosis when such sequelae persist for over two months following lithium cessation [13]. A review conducted in 2005 by Adityanjee et al. reported the typical clinical profile of SILENT to include persistent cerebellar dysfunction, extrapyramidal syndrome, brainstem dysfunction, or dementia [13]. Furthermore, atypical presentations of SILENT include retrobulbar optic neuritis, downbeat nystagmus, choreoathetoid movement, persistent papilledema, peripheral neuropathy, myopathy, and blindness [13]. In the presented case, the diagnosis of SILENT was proposed because despite improving lithium levels, the patient did not improve clinically. However, the patient’s clinical picture did not align with either the reported typical or atypical presentations of SILENT, and nor did the symptoms persist for two months, a necessary condition to qualify as SILENT. Despite that, SILENT should be among the differential considerations in such a presentation. Moreover, more studies of SILENT are needed to enrich the literature and explore its variants in more depth.

NMS was also considered a differential diagnosis in the presented case, as the patient was on antipsychotics. NMS is a clinical diagnosis that presents as a variety of symptoms, including fever, altered mental status, muscle rigidity, and autonomic instability [14]. Blood work can also show leukocytosis and elevated creatine kinase [14]. Current evidence suggests that NMS occurs less frequently during treatment with second-generation antipsychotics than with first-generation antipsychotics [15]. Due to favorable psychodynamics, second-generation antipsychotics were previously thought not to cause NMS [15]. However, physicians should know that NMS is a risk with all antipsychotics [15]. NMS was quickly ruled out in the presented case, as the patient was afebrile and vitally stable, with no autonomic instability or creatine kinase elevation [14]. There are reports of an association between NMS and lithium therapy combined with antipsychotics and an association between NMS and lithium toxicity [16,17]. Despite NMS being unlikely in the presented case, such a diagnosis should be included among the differentials when the patient takes antipsychotic medications. The clinical picture of NMS induced by quetiapine seems similar to “typical” presentations of NMS. In contrast, “atypical” NMS presentations were observed more frequently with clozapine-associated NMS (smaller in number extrapyramidal symptoms) and with aripiprazole-associated NMS (less severe autonomic symptoms and fever) [15].

SS was considered another differential diagnosis in the presented case. SS is a potentially life-threatening syndrome associated with increased serotonergic activity in the peripheral and central nervous systems [18]. It is seen with adverse drug-drug interactions, intentional overdose, and therapeutic medical use [18]. It is characterized by a spectrum of neuromuscular, autonomic, and cognitive dysfunction [18]. The patient in the presented case was previously on three different second-generation antipsychotics, all of which have 5-HT2A antagonistic properties, and lithium with 5-HT1 receptor activation properties [18]. Moreover, lithium toxicity can mask the presentation of SS [19]. Nonetheless, when taken in combination with other serotonergic medications, lithium can precipitate SS, as it can induce serotonin synthesis, increasing serotonin levels in cerebrospinal fluid [20]. There is also an overlap between the clinical manifestations of SS and lithium toxicity, as both can present with similar neurological and neuromuscular manifestations of altered mental status, myoclonus, hyperreflexia, and tremor, as in the present case [18,21,22]. Differentiating between the two conditions is essential, as management depends on the cessation of the offending agent and stabilizing the patient’s condition [23,24]. Lithium toxicity can necessitate hemodialysis, making it essential to differentiate it from SS; hemodialysis plays no role in managing SS [11]. The treatment team’s clinical judgment was that SS was less likely in the presented case.

Concerning the role of COVID-19, there are several reports showing that lithium toxicity is thought to be exacerbated by a severe acute respiratory syndrome coronavirus 2 (SARS‑CoV‑2) infection [25,26]. Patients in the reports commonly presented with altered sensorium due to lithium [25,26]. SARS-CoV-2 is thought to adversely affect kidney function, thereby affecting lithium excretion and precipitating toxicity [26]. Monitoring lithium levels in the context of a SARS-CoV-2 infection is therefore advised [26]. In the presented case, the SARS-CoV-2 infection is thought to have been acquired during the hospital stay, as the patient tested negative twice before testing positive on the sixth day following admission. Therefore, it is not likely that the infection played a role in the patient’s initial presentation.

Lithium can induce confused states by direct toxicity, by mediating the toxicity of other medications to produce NMS or SS, or by precipitating NCSE, as in the presented case [27]. By the third day following the patient’s admission to the medical ward, EEG imaging indicated NCSE. Effective termination of the seizures was established, and yet the patient did not improve clinically. Failure to improve could be attributed to various causes: post-ictal state, the side effects of ASM, or underlying encephalopathy.

Patients on lithium or other neuroleptic medications or with other medical conditions can pose diagnostic challenges to physicians in presentations similar to the current case [27]. Mental status changes, myoclonus, rigidity, movement disorders, and triphasic waves on EEG can be seen in multiple conditions, including NCSE, NMS, SS, and non-ictal encephalopathy, all of which can also be associated with lithium [27]. Triphasic waves on EEG imaging can usually be seen with metabolic encephalopathies, lithium toxicity, NMS, or SS, which can all resemble NCSE [27]. In patients treated with lithium, the EEG is a valuable tool in distinguishing non-ictal encephalopathy from ictal disorders and following up on patients’ clinical course, as lithium levels improve in cases of multifactorial encephalopathy [27]. In all the aforementioned conditions, namely, lithium toxicity, NMS, SS, metabolic encephalopathy, and NCSE, the EEG background may show slow and triphasic waves; however, a clinical and EEG response to benzodiazepine treatment is only present in cases of NCSE without encephalopathy [27,28]. Thus, Kaplan et al. recommend differentiating definite NCSE based on response to benzodiazepines [27].

There is also the possibility of an encephalopathy underlying the NCSE, which would explain why the EEG improved after starting the ASM but the patient’s condition did not improve [27]. Furthermore, there have been reports of encephalopathy, specifically valproic acid (VA)-induced HAE, as an adverse effect of the ASM [29,30]. In our patient’s case, further investigations revealed high ammonia levels, which could suggest the possibility of a metabolic encephalopathy underlying the NCSE. VA can induce various adverse effects involving the neurological, hepatic, hematological, and digestive systems [31]. VA-induced HAE is typically characterized by cognitive slowing, disorientation, delirium, focal neurological deficits, and an impaired LOC, which can range from drowsiness to coma, making it an important clinical consideration [32-34]. L-carnitine administration is suggested in cases of VA-induced HAE, as it has been shown to accelerate ammonemia normalization [35]. Carnitine supplementation tends to normalize ammonia concentration by binding to VA and reducing the inhibition of urea synthesis [35]. In our case, prompt management with L-carnitine and lactulose was initiated. Within a fortnight, the patient’s LOC and GCS improved, returning her to baseline.

In summary, in the presented case, despite normalizing lithium levels, the patient’s condition continued to deteriorate. The possibility of SILENT was initially proposed, and further imaging revealed the presence of NCSE. Although the seizure was successfully terminated, the patient’s LOC continued to deteriorate. The treatment team deemed NMS and SS to be less likely causes of her presentation, although these conditions were considered differential diagnoses. The patient’s laboratory results showed high ammonia levels. Following the correction of ammonia levels, the patient’s LOC finally improved.

This case report delineates how lithium toxicity could have a variety of sequelae, how it could entail several differential diagnoses, and how it poses management challenges for physicians in clinical presentations similar to this patient’s. This paper, like any other, has particular strengths and limitations. One of its strengths is that there have been no prior reports of similar cases in Saudi Arabia, emphasizing the need for further studies in the region exploring similar clinical presentations to better prevent, recognize, and treat patients to achieve the best clinical outcomes. Another strength of the paper is that it raises important questions for future consideration. For instance, it raises the question of whether, in similar cases, lithium as well as antipsychotics should be reintroduced. Indeed, the answer to this question should take multiple factors into account, including the patient’s age, the stability of the mood illness, and the need for monotherapy versus polytherapy, among other factors. On the other hand, as this case report only discusses the presentation of one patient, generalizing its results might be challenging. Hence, more research addressing similar presentations on a broader scale with a more rigorous research methodology is needed. Moreover, it is important to mention that other potentially contributing factors to the patient’s presentation were not excluded. For instance, the presence of anticholinergic toxicity as a differential and the patient’s pre-existing health conditions, such as dihydrolipoamide dehydrogenase deficiency, non-alcoholic fatty liver disease, and cholelithiasis, could all play a role in such a case.

## Conclusions

In this paper, we present and discuss a case of lithium toxicity, with the treatment team considering several differential diagnoses. The case highlights the complexity of the clinical presentation of lithium toxicity in some cases. Such a complex clinical presentation could entail extensive differential diagnoses, some of which could occur concurrently or even synergize with each other. Furthermore, such complexity imposes diagnostic and management challenges for the medical treatment team. Nonetheless, lithium toxicity alone can lead to several complications, some of which could be confused with the probable precipitating factors for it.

Prevention is always better than cure, and so early recognition of lithium toxicity, its possible precipitant(s), and its complication(s) is crucial. Such a healthy practice, namely, early recognition and intervention, would ensure the best medical outcomes for patients. Moreover, certain differentials, such as SILENT, are yet to be studied sufficiently to appreciate their clinical variations. Furthermore, physicians should treat each case comprehensively, considering patients’ clinical presentation, labs, imaging, and lithium serial level.
